# Positive Effects of Digital Technology Use by Adolescents: A Scoping Review of the Literature

**DOI:** 10.3390/ijerph192114009

**Published:** 2022-10-27

**Authors:** Aaron Haddock, Nadia Ward, Rondy Yu, Nicole O’Dea

**Affiliations:** 1 Frances L. Hiatt School of Psychology, Mosakowski Institute for Public Enterprise, Clark University, Worcester, MA 01610, USA; 2 School of Education, University of California, Riverside, Riverside, CA 92521, USA

**Keywords:** adolescent, development, digital technology use, positive outcomes

## Abstract

This study examines the research literature published from 2012 to 2022 on the relationship between increases in adolescent consumption of digital technologies and its impact on multiple areas of development, with a focus on how adolescent immersion in an increasingly ubiquitous digital world engenders positive outcomes in terms of brain, cognitive, and social-emotional development. The literature search yielded 131 articles, 53 of which were empirical studies of the relationship between increases in consumption of digital technology and brain development, cognitive development, or social-emotional development among adolescents. Overall, these studies identify positive outcomes for adolescents who use different types of digital tech, including the internet, social media, and video games.

## 1. Introduction

Today’s youth are growing up in a world in which digital technology is ubiquitous and integrated into nearly every aspect of life. Basic human activities, including those related to education, socialization, and recreation, increasingly take place on digital platforms which have spawned new modes of engagement (e.g., socialization via social media, recreation, and learning via video gameplay). According to a recent research report based on a nationally representative survey among a random sample of tweens (8- to 12-year-olds) and teens (13- to 18-year-olds) in the United States, digital media use among teens, which varies across multiple demographic variables (e.g., gender, race/ethnicity, and household income), is on the rise, up nearly 17 percent since the onset of the COVID-19 pandemic [1]. It is estimated that, on average, adolescents today spend roughly eight and a half hours a day engaged with digital media, not including their use of digital technology for schoolwork [1]. The largest increases in digital media use have been in watching online videos, using social media, and browsing websites. Of these activities, both tweens (8- to 12-year-olds) and teens (13- to 18-year-olds) report that watching videos on YouTube is their favorite form of digital media activity, followed in order of preference by Snapchat, TikTok, Instagram, Discord, Facebook, Twitter, Pinterest, Reddit, and Tumblr [1]. On average, teens spend close to an hour and a half a day on social media [1]. Around a quarter of teens play video games on a console or computer daily, but nearly half report playing mobile games daily [1]. In terms of time spent, teens spend the most time watching videos, followed by gaming on various platforms, social media, and browsing websites. In terms of gender differences, boys use more screen media than girls and enjoy video games more; girls enjoy social media more than boys do. In a nationally representative sample of 743 teens in the United States, 97 percent of boys said they play video games compared to 83 percent of girls [2]. About 20 percent of teens regularly listen to podcasts. In the 21st century, digital engagement via various technological devices, platforms, and tools has become necessary for youth to accomplish key developmental tasks.

The saturation of the environment with digital media has prompted adjustments to established theoretical paradigms and birthed the field of media ecology, which examines how interactions with technology in the media environment shape, affect, facilitate, and impede human development. Importantly for this review, media ecology looks specifically at the impact on adolescent development when key developmental activities and interactions are mediated by digital technologies [3,4]. To accomplish this, media ecology draws on research in developmental psychology and Bronfenbrenner’s bioecological model of human development, a foundational paradigm for the fields of developmental psychology, applied psychology, pediatrics and childhood studies [5,6].

The bioecological model of human development views individuals as biosocial beings placed at the center of nested systems that reciprocally interact to inform developmental outcomes [6,7]. At the core of this theory is the focus on proximal processes, or the reciprocal interactions between the developing individual and persons, objects or symbols within the immediate ecological context [8,9]. Human development is thus characterized as a product of the transactional relationship between the developing individual as an active agent and drivers of development across ecological contexts [10]. Bronfenbrenner’s bioecological model consists of five nested systems. The microsystem is the immediate environment in which the youth lives and includes any immediate relationships or organizations they interact with (i.e., caregivers, other immediate family members, school, or other places of care). Surrounding the microsystem is the mesosystem, which is essentially the different parts of the microsystem working together for the sake of the youth. For example, the mesosystem captures the interrelationships between the technologies youth engage with across home and school contexts. The exosystem includes other individuals and places that youth may not interact with directly but still influence development, including caregivers’ workplaces, extended family members, and the larger community context. Further, the macrosystem or outermost system of this model embodies sociocultural factors and ideologies that inform the ways in which youth development is supported across contexts [10]. This might include perceptions of tech engagement and misconceptions about influence that in turn dictate the extent to which youth engage with tech in the first place. Finally, the outermost system, or the chronosystem, captures the historical development of each system and the developing youth over time. This system is particularly important to consider given the historical advances in tech and the shifting discourse on digital tech effects on youth development.

**Figure 1:**
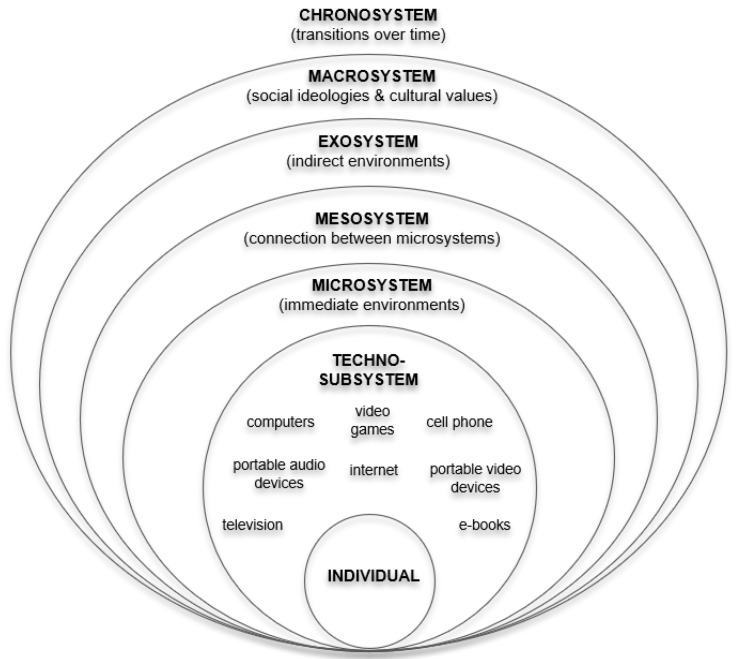
Ecological Techno-Subsystem.

Scholars are now updating the original bio-ecological framework to reflect how digital technology’s deep impingement into the microsystem and mesosystem impacts human development [11,12]. For example, Johnson and Puplampu [13] introduced the concept of the ecological techno-subsystem (see Figure 1). As a feature of the microsystem, this subsystem accounts for different types of technology and the interactions they support between the developing individual and others in their system (i.e., family, peers, teachers). This theoretical shift utilizes an ecological perspective to hone in on youth development while drawing from media ecology [14]. Media ecology focuses on the ways in which all types of media shape the psychosocial characteristics of individuals, recognizing the environment that media technologies provide for interaction and identity development [12,14]. Like the intent of this scoping review, a major question stemming from media ecology is how and why various forms of digital engagement facilitate or impede processes of development and in turn, developmental outcomes. Focusing on media ecology as part of the innermost nested subsystem of influence, the role of technology use in development becomes a critical element of consideration that warrants holistic exploration [12].

As a generation known as “digital natives”, [12,15] youth have choice and control in the type and frequency of which they engage with technology across ecological contexts that is unparalleled. The need to account for an additional zone that “mediates [the] bidirectional interaction between the child and the microsystem” in the most immediate developmental context underscores the profound influence of digital technology on child development in the 21st century.

### The Current Study

There is a growing body of research literature that identifies positive outcomes for youth who use different types of digital technologies, including the internet, social media, and video games. This study provides a scoping review of the extant literature examining adolescent consumption of digital technology and its impact on brain, cognitive, and social-emotional development, with a particular focus on how their immersion in an increasingly ubiquitous digital world engenders positive outcomes across these outcomes of interest.

## 2. Materials and Methods

In keeping with the research literature on digital engagement and media effects, this literature review employs the concept of digital media as a superordinate term that encompasses the broad category of types of digital technologies, applications, devices, platforms, and tools. Information and communication technologies (ICT) is another term frequently found in the literature that is synonymous with digital media. Similarly, Crone and Konijn [16] simply use the term media to describe the “media-saturated world, where media is used not only for entertainment purposes, such as listening to music or watching movies, but is also used increasingly for communicating with peers via WhatsApp, Instagram, SnapChat, Facebook, etc”. (p. 1).

This literature review employs the term digital engagement to capture youth’s “quotidian digital and online activity” and “the digital world”. Like digital media, digital engagement is “a broad concept of digital participation, which is not dependent on a specific technological device, platform, or tool” [17] (p. 102). An important aspect of adolescents’ and young adults’ digital engagement is captured by the concept of socio-digital participation (SDP) [18], which refers to participation in socio-digital activities via socio-digital technologies, defined as “the integrated systems of novel technological tools, social media, and the internet that enable constant and intensive online interaction with information, people, and artifacts” [19] (p. 16). Importantly, social-digital engagement is conceptualized as a participatory social practice reflective of adolescents’ lived experiences—and not merely acts of technology usage [19,20,21]. Typically, adolescents’ digital engagement activities are friendship-driven, interest-driven, or a combination of these digital engagement practices [21].

### Search Strategies

PRISMA is an evidence-based minimum set of items for reporting in scoping reviews, systematic reviews, and meta-analyses. In alignment with the PRISMA guidelines [22], the authors conducted a scoping review to source all literature with relevance to technology engagement and youth development. Articles were identified for possible inclusion from five relevant databases PsycINFO, PubMed, Google Scholar, PLoS, and PsychARTICLES; additional searches were conducted using Academic Search Premier, a large database that includes 8500 journals that cut across a range of scientific disciplines. Search terms included: Adolescen*, brain development, cognitive development, college and career readiness, communication skills, digital media, digital technolog*, learning, neuroplasticity, social development, social emotional, technology, youth. All searches included one search term related to technology (i.e., digital technolog*, digital media, technology) and a term related to a developmental outcome of interest (e.g., brain development, cognitive development). Terms were combined using AND when searches were intended to be inclusive of all terms (i.e., adolescen* AND digital technolog* AND cognitive development), while terms that can be interchanged were combined using OR (e.g., adolescen* OR youth). Results were limited to articles that were peer- reviewed and published between 2012–2022.

## 3. Results

**Figure 2:**
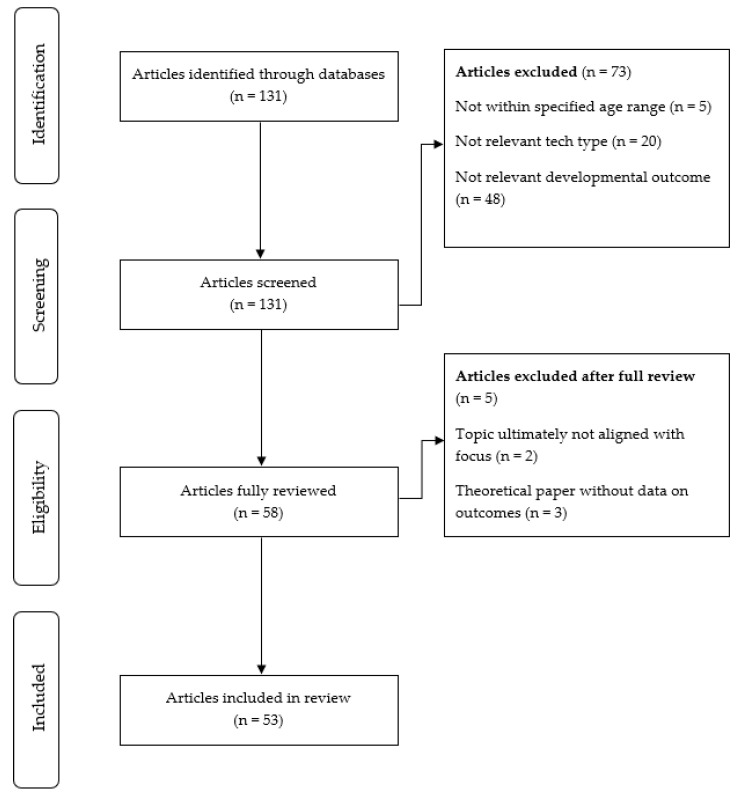
PRISMA Flow Diagram for Scoping Review.

The authors conducted an initial screening of all identified articles using the following inclusion criteria: (a) empirical study or review of the literature, (b) examines the effects of the use of digital technologies (i.e., internet, social media, video games) on at least one developmental domain of interest (i.e., brain development, cognitive development, social-emotional development, mental health/well-being). The initial search yielded 131 articles, of which 73 were excluded due to the criteria described above (see Figure 2). Fifty-three articles were fully reviewed between three of the authors. Inclusion decisions were made using a consensus approach where each article was discussed between at least three authors in a group format and then determined by the group to be included or not. Table 1 provides a summative overview of the selected articles organized by developmental domains of interest.

## 4. Findings

The discourse on the impact of digital media on youth is an extension of an age-old cultural concern and debate over the impact of new forms of technology on youth [23]. As Orben [24] has traced, concern and, at times even panic, over the influence of technology on youth has a long history. For example, in the Phaedrus, written circa 370 BCE, Plato recorded Socrates’ concern that the invention of writing and reading would ruin young people’s ability to use their memory and make them seem well educated and wise when in fact they were ignorant and unwise. In more recent centuries, tech fears have ranged from the novel giving rise to reading addiction, reading mania, and risky, immoral behavior in the 18th century to concerns about the negative influence of radio, television, smartphones, video games, and social media in the 20th and 21st centuries.

Although research on media effects has established that youth’s engagement with digital media can drive both positive and negative outcomes [3,25,26], the public perspective has focused more on its potential harm than benefits. Despite the focus on the negative impacts of technology on child development, the evidence linking digital engagement and negative outcomes is frequently overstated, focused on extreme users, and supported by studies lacking requisite nuance and complexity to discern specific effects [27,28]. Since the literature is largely based on correlational self-report data instead of sophisticated experimental designs, the direction of effects between digital media use and negative outcomes remains unclear [29,30]. When factors such as the type and quality of digital engagement, the social and developmental context, age, and individual differences are taken into consideration, digital engagement can function as a resource or a demand [31]. While it is a commonly held belief that digital engagement displaces important alternate activities, like sleep, interacting with friends and family, reading, and physical activity, the extant research has not substantiated this concern [27,32,33,34]. Conversely, the empirical evidence indicates that digital media facilitates peer communication, connection, and closeness (e.g., Davis [35]) and that engagement with tech at moderate levels is likely not deleterious [26,36,37] and may be promotive in a digital world (e.g., Giovanelli et al. [38]). For example, Lenhart et al. [39] found that social media use and collaborative gaming can facilitate friendships, social engagement, positive peer relations, and the provision of social support.

Digital media use is, according to Giedd [23], “in fact exquisitely aligned with the biology of the teen brain and our evolutionary heritage” (p. 128). Grounded in research on the neurobiological changes occurring during adolescence, Giedd clarifies how teens’ digital engagement is driven by changes in the brain’s reward system during puberty (dopamine, serotonin, GABA), teens’ efforts to accomplish key developmental tasks (e.g., Borca et al. [40]), and core features of the developing brain. fMRI studies on the adolescent brain demonstrate that, during adolescence, forming social connections becomes particularly salient and highly rewarding, which is reflected in their sensitized socio-affective brain circuits (Somerville, 2013). Given humans’ evolutionary history and the importance of strong connections with others, teens experience an existential drive for human connection, acceptance, and identification with groups (e.g., Crone & Dahl [41]; Blakemore & Mills [42]). Similarly, our evolutionary psychology predisposes humans to explore the environment, seek out adventure, and master threats—especially during the adolescent years when all social mammals exhibit increases in sensation seeking and risk taking. Adolescents also find experiences that enhance their affective development, or their emotional capacity to experience, recognize, and express a range of emotions and respond to others’ emotional cues, particularly reinforcing [41]. Developing the skills and aptitudes needed to transition to adulthood is highly motivating and rewarding for teens; whether these experiences take place in environments that are real or simulated matters little to the teen mind (e.g., Przybylski et al. [43]). Teens also exhibit a strong desire for information driven by evolutionary survival pressures and the human brain’s need for massive amounts of data from the environment for maturation (i.e., brain plasticity) and improved decision making. Thus, when it comes to digital technologies, what adolescents seek and find especially rewarding are opportunities to (1) face and overcome challenges, (2) connect and identify with a group, (3) grow emotionally, and (4) gain immediate access to actionable information.

### 4.1. Digital Tech & the Brain

While the research linking technology use and changes in the brain is still in its infancy, studies are emerging that indicate that digital engagement may positively (and negatively) influence human brains and behavior. For instance, studies utilizing brain imaging techniques have documented how intensive digital engagement can lead to changes in the brains of children and adolescents and affect brain functions, such as cognition, language, and visual perception (e.g., Firth et al. [44]; Hutton et al. [45]; Winnick & Zolna [46]).

#### 4.1.1. Video Games

Several studies have examined the connection between playing video games and brain structure using structural magnetic resonance imaging (sMRI). In one such study involving 152 adolescent participants in Germany, Kühn et al. [47] found a positive association between the reported amount of time spent playing video games (of any type) and cortical thickness in the prefrontal areas of the left hemisphere (i.e., dorsolateral prefrontal cortex and frontal eye field). They concluded that the thickness of the dorsolateral prefrontal cortex was related to executive control and the thickness of the front eye field was related visual-spatial attention and visual-motor integration.

Additionally, some studies have employed functional magnetic resonance imaging (fMRI) to examine the connection between playing video games and brain activity. For example, Mosaila et al. [48] used fMRI scans to compare the performance of 167 adolescents and young adults in Finland, who varied in terms of how frequently they played video games, on a task with selective attention and working memory demands. Results showed that those who reported playing video games more frequently displayed enhanced working memory functioning and task-difficulty-dependent modulation in a network of frontal and parietal brain areas in both hemispheres.

#### 4.1.2. Social Media/Internet

Studies have also documented specific brain regions engaged to build and maintain online social networks that are different from those used for offline social networks along with changes in the cortical volume of the brain stemming from engagement with peers via social media. Kanai et al. [49] found that, among participants in England, variation in online social network size strongly predicted gray matter volume and density in particular regions of the brain associated with social cognition, including navigating social networks and maintaining positive peer relationships, but not areas associated with understanding others’ actions, intentions, and perspectives. Kanai et al. [49] also found that online social network size was associated with areas of the brain responsible for remembering name-face associations. While this study was unable to determine the direction of the relationship between brain structure and participation in online social networks and whether friendships drive observed brain changes, scholars have pointed to these findings as evidence of adolescents’ and emerging adults’ sensitivity to the interpersonal dynamics involved when engaging with peers on social media.

### 4.2. Digital Tech & Cognitive Development

Cognitive development is best defined as the processes through which individuals acquire and organize new information or knowledge in order to apply it to novel situations [50]. Youth cognitive development is a salient domain when considering technology engagement. Often, engaging with technology like video games involves developing and sustaining problem-solving skills [51] and honing in on skills that enhance spatial recognition [52]. Below, we summarize findings that emphasize a positive relationship between tech engagement and both problem- solving and spatial skill development.

#### 4.2.1. Social Media/Internet

Fitton and colleagues [53] examined the relationship between internet use and cognitive and psychosocial development among a cohort of adolescents in the United States (N = 128). Authors conducted semi-structured interviews to gather insight on youths’ use of technology, level of comfort engaging with it, and how they feel it influences their own development. Overall, technology was perceived by youth as an integral part of their daily lives and a positive influence on their development. Specifically, they emphasized noticeable increases in skills and competencies related to recognizing information that they need and finding it on their own. With that, they recognized enhanced abilities in acquiring knowledge and creative thinking.

#### 4.2.2. Video Games

Uttal and colleagues [52] conducted a meta-analysis of studies that focused on trainings that aimed to improve spatial skills. Spatial skills of interest included: (1) spatial perception, or the ability to determine spatial relationships in relation to an individual’s own location even with distraction; (2) mental rotation, or the ability to visualize the movement of an object without any physical movement in order to make judgements; and (3): spatial visualization, or the ability to carry out a series of manipulations of stimuli that is spatially present [52]. Upon close examination of 217 studies involving diverse youth, authors concluded playing video games can be an effective training intervention to enhance spatial skills, where video game players across studies performed significantly better in tasks that require spatial attention and skill. Authors note, however, that the effectiveness of video game play as a spatial training intervention is based on personal characteristics, type of video game, and the duration and frequency of training sessions.

Kühn and colleagues [47] took a closer look at spatial skills by conducting a randomized comparative effectiveness trial with a sample of young adults in Germany (N = 48). The intervention arm, or video game training group, received instructions to complete various spatial tasks whereas the control group was instructed to freely explore during play. The training group engaged in video game training for at least 30 min a day for a span of two months using Super Mario 64, a widely known platformer game. Brain scans were conducted for both groups after the two- month training period. Results demonstrated significant differences between groups in brain imaging, showing an increase in gray matter in areas of the brain that are important for spatial navigation, strategic planning, and working memory. Overall, results supported the notion that video game training can be used to augment gray matter in the brain that are responsible for cognitive abilities.

### 4.3. Digital Tech & Social-Emotional Development

Social-emotional development is characterized by learning how to understand, manage, and express emotions in the context of learning about and building relationships with others [54]. Engaging with technology often involves a social context. Building on the social-emotional development literature, the Collaborative for Academic, Social, and Emotional Learning (CASEL) [55] has provided the most widely utilized definition of social and emotional learning (SEL): “SEL is the process through which all young people and adults acquire and apply the knowledge, skills, and attitudes to develop healthy identities, manage emotions and achieve personal and collective goals, feel and show empathy for others, establish and maintain supportive relationships, and make responsible and caring decisions”. Studies outlined below emphasize aspects of social-emotional development that are enhanced by tech engagement. For a research-based review of potential ways technology can be leveraged to support SEL (see Slovák & Fitzpatrick [56]).

#### 4.3.1. Digital Media

Przybylski and Weinstein [26] studied links between digital screen time (i.e., video games, computers, smartphones, films and other media) and mental well-being (i.e., happiness, life satisfaction, psychological functioning, and social functioning) in a sample of 120,115 15-year-olds in the United Kingdom. Female participants reported more engagement with smartphones, computers, and the internet, whereas male participants reported significantly more engagement with video games. Results indicated that moderate digital engagement (e.g., on a weekday, spending less than 1 h and 40 min playing video games or less than 1 h and 57 min using a smartphone) across device types is positively associated with mental well-being and does not appear to displace other activities that foster mental well-being. As the authors conclude, the study results suggest that, when used in moderation, digital technologies may “afford measurable advantages to adolescents” (p. 213), including providing opportunities for communication, creativity, and development.

It should be noted that the relationship between digital engagement and well-being among adolescents is still unclear and appears to vary by individual differences and the quantity and quality of digital media use. Studies have documented a variety of associations, including small, negative associations [28,57,58], no association [59], positive associations [60,61,62,63], and mixed results [64,65,66].

#### 4.3.2. Social Media/Internet

Studies have documented how adolescents’ social media use enhance social development and enhance relationships and social connections. For example, Reid et al. [67] found that social media platforms facilitate teens’ access to and interactions with others different from themselves, which increases understanding and empathy. In a study of 200 adolescents and emerging adults in Israel, Ziv and Kiasi [68] found that Facebook use provided users with a positive community that supported their psychological well-being; these effects were particularly pronounced for users with lower social skills who may have struggled more with in-person interactions. In a quantitative 7-day diary study of 162 adolescent Facebook users in Germany, Wenninger et al. [63] documented the positive association between targeted communication activities on social media that evoke reciprocity, like chatting and exchanging feedback via comments and likes, and positive emotions. As previously noted, this is in part because the adolescent brain is particularly sensitive to forming and maintaining social connections and developing an identity in relation to others.

#### 4.3.3. Video Games

Przybylski [69] examined the relationship between video game engagement and psychosocial adjustment (i.e., prosocial behavior, life satisfaction, and internalizing and externalizing problems) in a sample of 4899 10–15-year-olds from England, Northern Ireland, Scotland, and Wales. Analyses found small (<1.6% of variance) yet statistically significant positive associations between low levels of video game play and psychosocial adjustment. When compared to non-video game players, light video game play (i.e., less than one hour per day) was associated with positive psychosocial adjustment, including higher life satisfaction and prosocial behavior and lower levels of problems with peers, conduct problems, and emotional symptoms. No significant differences were detected for moderate levels of video game engagement (i.e., 1–3 h per day) when compared with nonplaying peers. However, heavy video game play (over 3 h daily) was associated with more negative psychosocial adjustment—indicating a possible dosage effect. Results suggest that playing video games responsibly provides youth with opportunities for socialization, identity development, and cognitive challenges that are facilitative of social-emotional development in a manner similar to more traditional forms of play.

In 2017, Adachi and Willoughby [70] reviewed the literature on the link between playing video games and positive youth outcomes, such as well-being, intrinsic motivation, learning, optimal functioning, and positive peer relationships. The review focuses on studies that apply self-determination theory (SDT) to explain how video games may create contexts that satisfy basic psychological needs (i.e., competence, autonomy, and relatedness) and, in turn, effectuate positive outcomes. Citing numerous studies published between 2000 and 2016, the authors argue convincingly that playing video games afford experiences of independence, interdependence, cooperation, exploration, and challenge that in turn foster enhanced autonomy, competence, human relatedness, and well-being. The review also establishes a link between playing video games and developing problem-solving skills (e.g., identify the problem, generate and evaluate possible solutions) that hold the potential to not only improve adolescents’ game play but also their peer relationships. This link is further buttressed by research on how playing online video games cooperatively with diverse youth enhances intergroup relations and feelings of social connection.

EmoTIC is an example of a game-based social-emotional program with demonstrated impact on adolescent social and emotional development [71]. The intervention has a science-fiction theme and is delivered via a digital app. Users participate in four classroom group sessions and complete twelve individual home activities focused on acquiring foundational SEL concepts (e.g., emotional skills, social skills, enhancing self-knowledge and self-esteem, and assessing growth. Results showed that adolescents in Madrid, Spain between the ages of 11 and 15 (*n* = 119) who completed the program improved on several measures, including self-esteem, feelings of well-being, emotion regulation, and prosocial behavior.

### 4.4. Digital Tech & Mental Health/Well-Being

Youth mental health and well-being is an all-encompassing term that represents a balance of emotional, psychological, and social wellness [72]. It involves the ways in which youth handle stress, practice healthy habits, and maintain social engagement. Mental health and well-being are particularly important for youth as they are at the cusp of developmental milestones that heavily rely on mental, social, and emotional wellness.

#### 4.4.1. Video Games

Video games possess the unique ability to enable adolescents to experiment with and “try on” different identities and experiences not available in their current life situation or developmental phase. In a study of emerging adults (nationality not provided) by Przybylski et al. [43], researchers found that when games facilitated alignment between players’ ideal-self characteristics and game-self characteristics, players experienced higher levels of intrinsic motivation and well-being. These results suggest that digital engagement experiences that enable adolescents and young adults to simulate and experience ideal aspects of themselves (e.g., helping others, graduating from college, having a desirable career) may enhance motivation to engage in the experience while offering virtual exposure experiences that promote self-exploration and goal identification and adoption.

Barr & Copeland-Stewart [73] examined video game play and youths’ overall well-being during the COVID-19 pandemic. Using an online survey with closed and open-ended questions, authors measured game play habits and aspects of mental health and well-being among a large sample of youth (N = 781) during the pandemic. Results indicated that youth engaged in more frequent game play for longer segments of time, describing their extended engagement as an “escape from the pandemic”. This finding correlated with increased socialization during the lockdown advisory and decreased anxiety and depression. Further, respondents reported feeling as though engaging in video game play provided more feelings of control and agency during a particularly challenging time. Overall, this article emphasizes that video game play can provide support and relief that contributes to improved mental, social, and emotional wellness.

#### 4.4.2. Social Media/Internet

Multiple studies have demonstrated that most people use social media to support, maintain, and enhance offline social relationships [74,75,76]. For instance, in a nationally representative survey of teens in the United States (743 youth between the ages of 13 and 17) by the Pew Research Center, 31 percent of teens said that social media has had a mostly positive impact, especially when it comes to connecting and staying in touch with friends and family [2,77]. Respondents also emphasized how their use of social media enabled them to meet and connect with others with similar interests, explore their identity and express themselves, garner peer support, and learn new things. Eighty one percent said social media makes them feel more connected to their friends; 69 percent said it helps them interact with a more diverse group of people; and 68 percent said it makes them feel as if they have people who will support them through tough times. Overall, teens associated social media use with positive emotions, including feelings of inclusion and confidence [2].

Kanai et al. [49] found that variability in the size of users’ offline social networks was correlated with variability in the size of users’ online networks. Building on this finding, Davis [61] examined the impact of digital media use and online peer communication on friendship quality in 2079 adolescents in Bermuda. Analyses revealed a positive association between more frequent online communications with friends and friendship quality. In discussing the findings, Davis noted that studies support the view that, despite the negative public perception, the existing evidence suggests that online peer communication is largely positive and serves to enhance peer relationships.

In a systematic review of the literature, which included large numbers of adolescents and emerging adults, Erfani and Abedin [78] found social media use led to increased well-being and had positive effects on users’ social support, communication, and connectedness. Meta-analyses have also found that connecting with others via social media enhances both social support and users’ perceived social resources [79,80].

There are some potential limitations concerning the results of this study. It is possible that the search terms used were not inclusive of all possible variants and the databases searched were not inclusive of all relevant journals, thus resulting in the exclusion of relevant studies. However, it is important to note that scoping reviews are not intended to be exhaustive [81]. In addition, it is possible that the synthesis literature included in this review suffers from the prevalent issue of non-independence of observations (i.e., overlap among primary-level studies). However, non-independence “may be fairly minimal” in reviews that draw from a broader body of literature that includes sources representing many different disciplines [82].

### 4.5. Conclusions

In sum, this scoping review of the empirical research literature on the relationship between digital engagement and positive youth development found evidence of specific positive effects on adolescent brain development, cognitive development, social-emotional development, and mental health and well-being. These included improvements in executive control, visual-spatial attention, visual motor integration, problem solving, working memory, strategic planning, and information gathering; increases in social-emotional learning, intrinsic motivation, socialization, social support, social connection, and creativity; and enhancements to autonomy, competence, communication skills, and well-being.

### 4.6. Recommendations for Leveraging Digital Tech Use to Promote Positive Outcomes for Adolescents

Given the documented impact of digital engagement on adolescent development, tech-based interventions demonstrate promising potential across domains of youth development. There is clearly a unique opportunity to leverage technology in a manner that will positively engage teens and intervene with them to help them learn about themselves, advocate for themselves, and explore careers. However, as evidenced by the scoping review, there is a limited number of articles that focus primarily on positive outcomes. The following recommendations are based on the findings of the scoping review, behavior change design principles, and insights from startup product development.

#### 4.6.1. Employ an Intervention Design Process

When developing tech-based interventions, begin by carefully defining the problem to be solved, the outcomes of interest, the target users, and the target users’ relevant contexts. Then, explore a variety of potential solutions. When exploring solutions, consider (a) possible intervention designs and (b) possible tech-based delivery methods. The findings of this literature review are particularly pertinent to this phase of the design process and will help clarify which types of solutions are likely to be most effective. This can be visually represented in an outcome logic map or logic model. Program evaluators employ logic models to define the specific outcomes an intervention is intended to achieve, the activities (i.e., mechanisms of action) that will facilitate achievement of the targeted outcomes, and how the intervention’s results will be measured. When applied to tech-enabled interventions, it is especially important to clarify how the intervention will be implemented and used.

Employ a customer discovery approach to determine how best to meet potential users’ needs [83]. Interview target users to understand their perspective, motivations, priorities, values, goals, and identities. Explore their reasons for engaging with the intervention being developed. Ask them what tech product features and intervention components they think will help them achieve the target outcomes (e.g., increased self-knowledge, career exploration). If the intervention aims to change behavior, consider using the Behavior Change Wheel as a product design framework. Based on in-depth research on 19 behavior change frameworks, the Behavior Change Wheel helps product designers identify solutions that enhance users’ capability, opportunity, and motivation to change or engage in a particular behavior. See Michie, Atkins, & West’s practical guide to intervention design, The Behaviour Change Wheel: A Guide to Designing Interventions [84] and Bucher’s Engaged: Designing for Behavior Change [85].

#### 4.6.2. Convene a Youth Advisory Board

If teens are the target users, engage teens at every phase of the design process to create a teen-centric intervention that connects their motivations with the target outcomes. Convene a diverse and inclusive teen advisory board to get their perspectives and solicit their guidance on what teens need, want, and will use. Once a beta version of the intervention is ready, relentlessly collect feedback from teens on what works and what needs to be changed.

Have the teen advisory board work closely with subject matter experts and technologists to ensure the interventions and experiences integrate into their lives and use language they will respond to. Like other popular forms of digital engagement, interventions will work best if they meet youth where they are at in familiar and fun ways.

Highlight teens involvement with the creation of the intervention and provide opportunities for teens to promote it.

#### 4.6.3. Create Authentic and Engaging Digital Experiences

Youth are particularly sensitive and responsive to authentic social media messaging. Social media campaigns and initiatives will be most effective if developed and deployed by youth with the support of subject matter experts. Rallying authentic youth engagement (e.g., “likes”, “retweets”, etc.) and promotion of the campaign or message is key. For example, a social media campaign aimed at inspiring youth to consider pursuing a technical career as a possible alternative to college could begin by convening a teen advisory board charged with discovering, for example, teens’ questions about the decision to pursue a technical career. The youth advisory board’s process of gathering this information (e.g., through social media queries, focus groups, surveys, interviews, etc.) could be shared in creative social media posts, videos, and photos and serve to promote the campaign and create a community around the initiative online. This will build trust and buy-in.

Digital interventions that leverage social media should take into consideration the social norms of the platform. There is evidence to suggest that teens are increasingly reluctant to explore, experiment with, and express their identity or emerging identities on mainstream social media platforms like Facebook and Instagram, but continue to do so on YouTube and fanfiction sites and in microblogging communities [86]. Therefore, interventions that aim to facilitate identity exploration and development in the service of greater self-awareness and self-knowledge should keep this in mind and create online spaces where teens feel understood, experience camaraderie, and can be genuine and engage in authentic self-exploration [61,87]. Ideally, an online social network will engender positive growth, provide teens with social support, and connect them with the peers, experts, and professionals that will help them achieve their goals and the intervention’s target outcomes.

Tech-based interventions to facilitate self-knowledge, self-awareness, and identity development should provide youth with opportunities to explore different identities, including idealized versions of themselves, and contexts that are not currently accessible. For instance, a video game or virtual reality experience could enable users to try out different careers in a variety of roles (e.g., programmer, team leader, copywriter, marketing director). An app could help teens imagine their future self in college or a career coupled with an opportunity to set short- and long-term goals and create a detailed action plan aligned with their values. The app-based action plan could guide and support users as they take concrete steps toward their goals, offer timely tips and encouraging feedback, and celebrate and reward users when they reach important milestones on their journey.

If the digital intervention leverages a social component, provide users with choice. The research is clear that online social support can be beneficial for teens; however, not all users want to engage in a social aspect or have their activities be made public. Nevertheless, provide all users with the option to witness the social engagement of others, even if they do not participate. Research has shown that witnessing the online social engagement of others can be nearly as beneficial as active participation [85].

#### 4.6.4. Leverage the Best Features and Most Popular Forms of Digital Engagement

Teens range freely across digital media platforms and tools. Design tech-enabled interventions that leverage the best features of gaming, social media, online videos, streaming, and digital content creation devices. Take teens’ favorite forms of digital media activity into consideration; among teens in the U.S., research indicates watching videos on YouTube is their favorite form of digital media activity, followed in order of preference by Snapchat, TikTok, Instagram, Discord, Facebook, Twitter, Pinterest, Reddit, and Tumblr [1].

Leverage technological innovations to support SEL. For example, use mobile devices to provide youth with just-in-time prompts (e.g., to label emotions and practice emotion regulation skills when high levels of stress are detected by physiological sensors), reminders (e.g., to engage in activities that promote well-being or facilitate social connection), and scaffolding and support (e.g., how to use problem solving skills; prompts to track experiences to facilitate self-reflection and/or discussions at a later time). Use social media sites to support reflection, sharing of experiences, and social-emotional self-awareness. Online social networks can also be used for online support groups that provide, for instance, information on and support around college and career exploration or mental health. Natural language understanding technology is already being used by mental health professionals to monitor therapy sessions and glean evidence-based insights; a similar approach could be applied in everyday life to foster social-emotional development and communication skills.

While virtual reality’s (VR) ability to create powerfully immersive experiences continues to hold incredible promise for SEL, skill building, and mental health promotion and treatment [88], currently less than 20 percent of youth have access to VR headsets [1].

#### 4.6.5. Use Video Games to Build Community, Provide Exposure Experiences, Explore Identity, and Enhance Perspective Taking

Video game play among youth is particularly high and thus provides a unique opportunity to engage their interests in a way that promotes development and engages them within the community. Offering opportunities for youth to engage in game play within the community, for example, in-person or virtual tournaments, provides youth with a platform that not only supports their cognitive development and psychosocial wellness, but also maintains social connection during the extended pandemic period. There is also evidence to suggest that interactive media experiences can facilitate perspective taking, communication skills, and collaboration.

When designing video game-based interventions, provide the player with enough challenge to make the game engaging, but not so challenging that the player feels the task is insurmountable. Game-based interventions that provide players with novel opportunities to embody and experience ideal aspects of themselves (i.e., how they would like to experience themselves) will enhance intrinsic motivation to play the game along with enjoyment [43].

#### 4.6.6. Understand the Environment in Which Digital Interventions Are Implemented

Considering how technology now mediates interactions between the developing individual and others in their microsystem (i.e., family, peers, teachers), when designing tech-based interventions it is important to consider the role it will play in teens’ techno-subsystem. For example, how will this intervention integrate with what teens are already doing in their daily lives? How may the reciprocal interactions with other important individuals in the teens’ microsystem facilitate or impede intervention effectiveness? How might influential peers, parents, and mentors be recruited to support and amplify the aims of an intervention? Tech-based interventions that easily integrate with existing influential relationships in the youth’s microsystem will be most engaging and effective.

In light of changes in the brain occurring during adolescence, the most naturally engaging and effective digital interventions will: (a) be fun, engaging, and social; (b) foster emotional growth; (c) give teens agency over their education; (d) enable identity exploration and experimentation; (e) engage other influential people in teens’ developmental context; (f) help teens draw connections between their core values, priorities, and short- and long-term goals, (g) empower exploration and mastery of their environment; (h) facilitate achievement of key developmental tasks; and (i) provide immediate access to actionable information.

## Figures and Tables

**Table 1:** Overview of Selected Articles.

Developmental Domain	Articles(N = 53)	Tech Types	Major Findings
Brain	6	Video games	Positive association between time spent playing video games and increases in volume of gray matter in various parts of the brain (e.g., hippocampus, prefrontal cortex) Positive association between time spent playing video games and increases in performances in tasks requiring selective attention, working memory, and visual-motor integration
Cognitive	11	Video games, Internet use	Video game training augments gray matter in areas of the brain that relate to cognitive abilities related to spatial navigation, strategic planning, and working memory. Video games help balance cognitive stimulation and exercise the brain. Internet use contributes to task completion and skill competencies.
Social-Emotional	22	Video games, Internet use, Social Media	Video games promote mental “healthiness”, support perceived control and agency, and helps to foster initiative in youth. Video games create contexts that can help satisfy basic psychological needs (competence, autonomy, relatedness) and, in turn, effectuate positive outcomes. Video games afford opportunities to virtually experience different identities and situations that can promote self-exploration and goal adoption. Prosocial gaming does not influence later executive function-specifically fluid reasoning and empathy, a related SEL function. Game-based SEL programs can increase social-emotional competencies. Internet use increases the ability to think creatively and reflect on social relationships. Social media facilitates social connection, identity development, and positive emotions. Online peer communication via social media enhances friendship quality, perception of social support, connectedness.
Mental health and well-being	14	Video games	Video games provide relief for stress and anxiety during periods of adversity (i.e., the COVID-19 pandemic). Video games promote a sense of connectedness and address feelings of isolation. Video game play at responsible amounts facilitates positive psychosocial adjustment (e.g., prosocial behavior).

## Data Availability

The data presented in the study is available upon request from the first author.
